# Community end-of-life care during the COVID-19 pandemic: findings of a UK primary care survey

**DOI:** 10.3399/BJGPO.2021.0095

**Published:** 2021-07-14

**Authors:** Sarah Mitchell, Phillip Oliver, Clare Gardiner, Helen Chapman, Dena Khan, Kirsty Boyd, Jeremy Dale, Stephen Barclay, Catriona R Mayland

**Affiliations:** 1 Yorkshire Cancer Research Senior Research Fellow, Department of Oncology and Metabolism, University of Sheffield, Sheffield, UK; 2 Clinical Lecturer, Academic Unit of Medical Education, University of Sheffield, Sheffield, UK; 3 Senior Research Fellow, Division of Nursing and Midwifery, Health Sciences School, University of Sheffield, Sheffield, UK; 4 Queen's Nurse and Head of Integrated Community Care, Sheffield Teaching Hospitals NHS Foundation Trust, Sheffield, UK; 5 Patient and Public Involvement Representative, University of Warwick, Warwick, UK; 6 Reader in Palliative Care, Usher Institute, University of Edinburgh, Edinburgh, UK; 7 Professor of Primary Care, Warwick Medical School, University of Warwick, Coventry, UK; 8 Senior Lecturer in General Practice and Palliative Care, Department of Public Health and Primary Care, University of Cambridge, Cambridge, UK

**Keywords:** primary health care, general practice, district nursing, palliative care, end-of-life care, COVID-19

## Abstract

**Background:**

Thousands of people in the UK have required end-of-life care in the community during the COVID-19 pandemic. Primary healthcare teams (general practice and community nursing services) have provided the majority of this care, alongside specialist colleagues. There is a need to learn from this experience in order to inform future service delivery and planning.

**Aim:**

To understand the views of GPs and community nurses providing end-of-life care during the first wave of the COVID-19 pandemic.

**Design & setting:**

A web-based, UK-wide questionnaire survey circulated via professional general practice and community nursing networks, during September and October 2020.

**Method:**

Responses were analysed using descriptive statistics and an inductive thematic analysis.

**Results:**

Valid responses were received from 559 individuals (387 community nurses, 156 GPs, and 16 unspecified roles), from all regions of the UK. The majority reported increased involvement in providing community end-of-life care. Contrasting and potentially conflicting roles emerged between GPs and community nurses. There was increased use of remote consultations, particularly by GPs. Community nurses took greater responsibility in most aspects of end-of-life care practice, particularly face-to-face care, but reported feeling isolated. For some GPs and community nurses, there has been considerable emotional distress.

**Conclusion:**

Primary healthcare services are playing a critical role in meeting increased need for end-of-life care in the community during the COVID-19 pandemic. They have adapted rapidly, but the significant emotional impact, especially for community nurses, needs addressing alongside rebuilding trusting and supportive team dynamics.

## How this fits in

This study provides insights into experiences of delivering end-of-life care in the community during the first wave of the COVID-19 pandemic from the perspectives of UK GPs and community nurses. Services have changed and adapted rapidly to meet increased need in terms of both volume and complexity. Community nurses have taken greater responsibility for most areas of palliative care clinical practice, and GPs have undertaken more care planning conversations. While GPs and specialist palliative care services conducted more remote consultations, community nurses carried out face-to-face end-of-life care and reported a feeling of isolation. As the pandemic progresses, and the increased need for end-of-life care in the community persists, more effective service models and multidisciplinary teamwork in primary care are urgently needed.

## Introduction

The COVID-19 pandemic has caused primary healthcare services (general practice, and community nursing services including district nurses) to dramatically change their traditional models of service delivery over a short timeframe. Palliative and end-of-life care for people at home was affected from the outset.^1–3^ The first 10 weeks of the pandemic saw a significant increase in deaths in the community, particularly in people aged >75 years. Deaths in care homes increased by 220% and deaths at home increased by 77%, while deaths in hospices fell by 20%,^4^ presenting significant workload in this area for primary healthcare teams. While guidance documents for both general practice and community nurses outlined end-of-life care as urgent priorities,^5,6^ there were many new challenges in the delivery of that care, including the need for more remote consultations and the use of personal protective equipment. Furthermore, there were concerns about drug and equipment supplies and the need to manage new symptom profiles associated with COVID-19.^7^ Previous research and policy guidance for primary healthcare services in pandemics do not tend to refer to community end-of-life care, and there was little evidence to inform and guide the necessary service changes.^8,9^

There is an urgent need to understand more about the role and response of general practice and community nursing services in the delivery of palliative care during the COVID-19 pandemic. This understanding is required in order to inform practice and policy through the next phases of COVID-19 and future pandemics.

### Aim

The study aimed to provide insights and understanding into the experiences and perceptions of primary healthcare professionals within the UK, who were providing palliative and end-of-life care in the community during the first wave of COVID-19.

## Method

An online questionnaire study was the most feasible method to meet the research aims, gathering both quantitative and qualitative data rapidly. The development of the survey instrument was informed by patient and public involvement, a literature review conducted by this research team,^9^ the CovPall study,^10^ and feedback from a study advisory group of clinicians and commissioners. The survey instrument was pre-tested by a group of 17 GPs and community nurses. As a result of their feedback, changes were made to aid clarity to the wording of individual questions and the order of the survey questions.

The survey contained open and closed questions. Demographic data were collected about the participants and the services they work in. Qualitative and quantitative data were then collected about specific aspects of community palliative and end-of-life care provision during the first wave of COVID-19. The survey was divided into sections regarding different aspects of end-of-life care provision, with a selection of open-response questions designed to capture important aspects or personal reflections from participants that were not captured elsewhere (Supplementary file S1). The STROBE checklist informed the reporting of the study.^11^

### Patient and public involvement

Patient and public involvement (PPI) was integral to this research, with a PPI co-applicant joining the research team and further PPI sought through the University of Sheffield Palliative Care Studies Advisory Group (PCSAG). Both the local PPI work and a national consultation exercise^12^ highlighted the importance of the provision of end-of-life care in the community during the COVID-19 pandemic. Group members provided comments on the literature review and assisted in the development of the research questions, survey instrument, and overall design of this research.

### Data collection

The survey was circulated via social media and UK professional networks, locally and nationally, including through the Royal College of General Practitioners, the Society for Academic Primary Care, the Royal College of Nursing, the Queen’s Nursing Institute, and the National District Nursing Network. Responses from GPs or community nurses were included, responses from other healthcare professionals were excluded. The target sample size was 500, with sample sizes in this region having previously been achieved.^13^ This was considered a large enough sample to capture diversity of experience in a short timeframe, although not necessarily representative of the wider primary care workforce. Data were collected between 1 September and 16 October 2020. All responses were anonymous.

### Data analysis

Quantitative data were analysed using descriptive statistics using SPSS (version 26). Responses to each item were analysed using a χ^2^ test to assess the association between role (nurse versus doctor) and response to the question, and to examine any evidence of a difference between these two groups in how they responded. Paired Z-tests were conducted to understand whether there was any significant differences between the specific items within the question. The percentages are presented with 95% confidence intervals.

Qualitative responses were anonymised, uploaded into NVivo 12 software (version 10), and analysed using an inductive, iterative thematic approach.^14^ For the purposes of this article, the qualitative findings relating to personal reflections about the provision of end-of-life care during the initial wave of the COVID-19 pandemic are reported. Codes were assigned to each data item, then all codes collated, mapped out, and compared to extrapolate overarching themes.

The initial data analysis was led by SM (qualitative), CM (quantitative and qualitative), and PO (quantitative) with regular discussion of the emerging findings in order to reduce lone researcher bias.^15^

## Results

### Demographics

In total, 563 responses were received; healthcare professionals outwith the target groups were excluded, leaving 559 valid responses. Of these, there were 387 community nurses, 156 GPs, and 16 did not specify their role. The latter group was included in summary descriptive statistics but omitted from any group comparisons or qualitative analysis (Table 1). Responders represented all countries within the UK with over three-quarters (77.1%) working in England. Different types of rurality were represented, with the largest proportion from a ‘mixed urban and rural’ area (*n* = 222, 39.9%). As the findings from the analyses emerged, it became clear that both revealed different experiences among community nursing staff compared with GPs. Quantitative and qualitative findings are, therefore, presented together in this section as three inter-related themes.

**Table 1.** Demographic details of responders and information about provision of care for dying patients during the pandemic (*n* = 559)

	***n***	**%**
**Which country do you work in?** (*n* = 559)		
England	431	77.1
Scotland	65	11.6
Wales	47	8.4
Northern Ireland	16	2.9
*Missing*	*0*	
**What type of area do you work in mainly?** (*n* = 556)		
Mixed urban and rural	222	39.9
Urban	179	32.3
Rural	106	19.0
Inner city	49	8.8
*Missing*	*3*	
**What is your role?** (*n* = 543)		
**Doctor**	**156**	**28.7**
GP partner	104	19.1
Sessional GP	45	8.3
Other, for example, GP in training	7	1.3
**Community nurse**	**387**	**71.3**
Community staff nurse	150	27.6
District nurse (including team leaders)	159	29.3
Advanced nurse practitioner	32	5.9
Community matron	24	4.4
Community healthcare assistant	15	2.8
Nurse consultant	7	1.3
*Missing*	*16*	
**Have you cared for any patients in the community who have died with confirmed (by test) COVID-19?** (*n* = 557)		
Yes	296	53.1
No	261	46.9
*Missing*	*2*	
**Have you cared for any patients in the community who have died with suspected COVID-19 (untested but with clinical symptoms)?** (*n* = 554)		
Yes	371	67.0
No	183	33.0
*Missing*	*5*	
**Have you been involved in providing end-of-life care at home for patients who do not have COVID-19 or suspected COVID-19 through the pandemic?** (*n* = 554)		
A lot more than usual	172	31.1
A little bit more than usual	150	27.1
About the same as usual	211	38.1
A little bit less than usual	13	2.3
A lot less than usual	8	1.4
*Missing*	*5*	
**Have your working hours changed in order to deliver end-of-life care during COVID-19?** (*n* = 554)		
Yes	189	34.1
No	366	65.9
*Missing*	*4*	

### Theme 1: Increased need, complexity, and unpredictability in community end-of-life care during COVID-19

Responders reported increased involvement in the provision of end-of-life care in the community as a consequence of the pandemic, with over half providing end-of-life care ‘a lot more’ or ‘a little bit more than usual’ (*n* = 322, 58.2%) to patients who were not known or suspected to have COVID-19. Over half of responders (*n* = 296, 53.1%) reported caring for patients who had died with ‘confirmed’ COVID-19, and over two-thirds (*n* = 371, 67.0%) reported caring for those who had died with ‘suspected’ COVID-19 (Table 1).

Working hours changed in response to the need, with these changes often arranged informally, and frequently unpaid:

*'Informal changes to work patterns, working well over shift times due to symptom management and workload also factoring in staff shortages due to COVID-19 and shielding.'* (Responder 214, community staff nurse, Northern Ireland)

An important factor was patients choosing to remain at home to receive end-of-life care rather than being admitted to hospital. This presented workload challenges, but was recognised as a long-term aspiration for patient care that was achieved because of the pandemic:

*'I feel more patients stayed at home for non-COVID related end-of-life care. Which was good. Think the staff that were at the front line went above and beyond to keep patients at home. Patients and families did not want admission as then they could not see family etc, and then die without family there. Staying at home was seen as best option for most patients and families, even if it was tiring.'* (Responder 55, district nurse, England)

Nurses highlighted an associated increase in the management of patients with more complex healthcare needs, which were not related to COVID-19:

*'We have had more complex patients being managed at home which has been a challenge, whereas if COVID-19 and visiting wasn't an issue they may have been hospice inpatients or even admitted to an acute hospital bed.'* (Responder 299, sessional GP, England)

Increased patient care needs resulting from the pandemic included an increase in frailty among those living with advanced illness who were shielding at home:

*'Patients within the community have shielded well but we are seeing declining health conditions due to shielding … more frailty has been identified during COVID-19 as patients have lost their daily routines and independence. Lack of exercise and carrying out their normal activities of daily living has resulted in more frailty.'* (Responder 483, district nurse team leader, England)

The care of patients with COVID-19, as a new condition (or suspected COVID-19 when testing was not available), was associated with a high level of unpredictability and clinical uncertainty, and a variety of presentations of dying. Only four responders reported patients dying with distressing symptoms owing to COVID-19 such as agitation, breathlessness, and abdominal pain. There were more than 100 accounts of experiences of providing care for a large number of patients who deteriorated and died very rapidly, particularly frail older patients, including those in care homes:

*'Before testing of clients I found that community and care home clients would be walking and healthy, then suddenly develop a temperature over 38 degrees* [centigrade] *and take to their bed, they would be lethargic and confused, not eat or drink and within approx 72 hours or so would have died.* *'* (Responder 142, community staff nurse, England)

### Theme 2: The roles of the primary care multidisciplinary team to meet increased demand in the context of COVID-19

**Figure 1.**
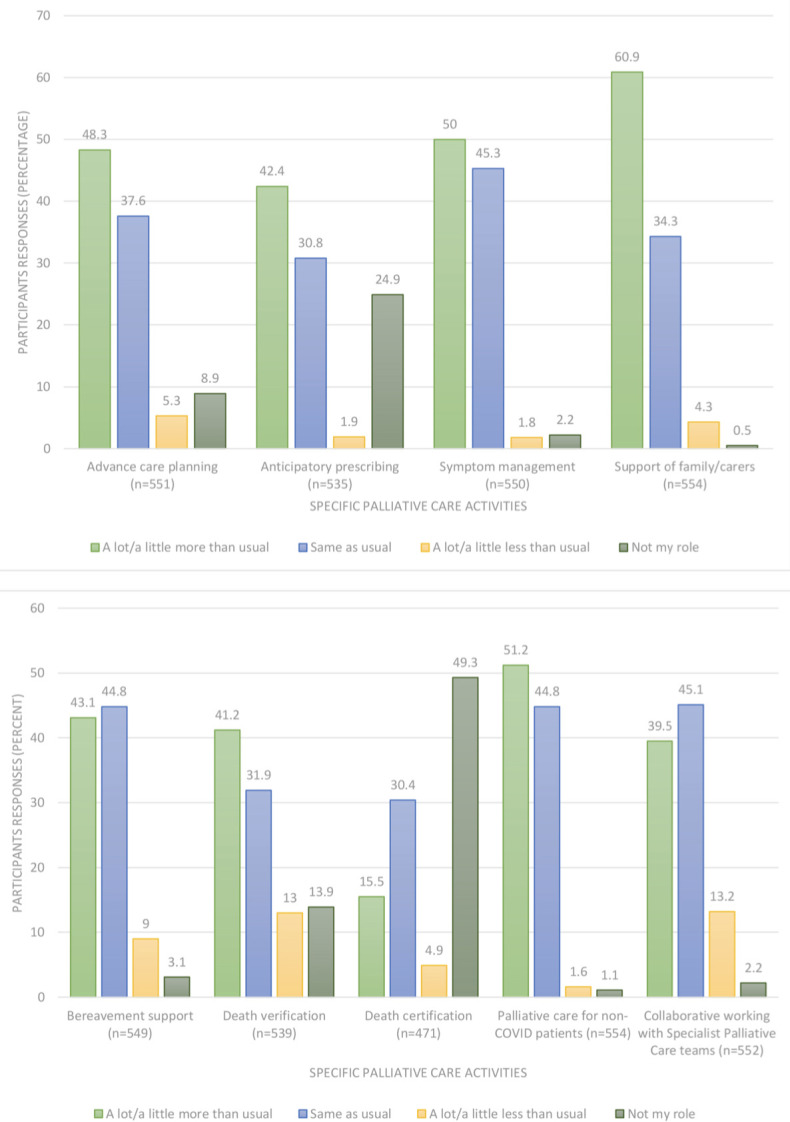
Changes in professional role in end-of-life care (*n* = 559)

There were rapid changes in the roles of members of the community multidisciplinary team (MDT) to meet the increased need for end-of-life care, with responders reporting that they were undertaking more advance care planning (48.3%), anticipatory prescribing of medication (42.4%), symptom management (50.0%), bereavement support (43.1%), and death verification (41.2%) (Figure 1). Additionally, 339 (60.9%) reported that they were providing support to family and carers ‘a lot or a little more than usual’. There was variation in teamworking activities with specialist palliative care teams, with 218 (39.5%) responders reporting increased collaborative activities whereas 73 (13.2%) reported fewer.

Comparing GPs’ and community nurses’ responses showed differences in their professional roles for palliative care activities (Table 2). GPs undertook ‘more or a lot more’ advance care planning compared with the community nurses (*P*<0.0001), while community nurses reported providing ‘more or a lot more’ symptom management (*P*<0.0001), bereavement support (*P*<0.0001), death verification (*P*<0.0001), palliative care for those without COVID-19 (*P*<0.0001), and collaborative working with specialist palliative care teams (*P*<0.0001). Although both groups indicated they had given more support for family and carers, a significantly larger proportion of community nurses answered ‘more or a lot more’ compared with GPs (66.0% versus 48.1%, *P* = 0.01).

**Table 2.** Comparison between community nurses' and doctors’ responses for their professional role in palliative care activities

	**Please state whether your role in the following areas of palliative care has changed during the COVID-19 pandemic^a^**
**Community nurses**	**GPs**	***P* value^b^**
**Total**	387	±95% CI (%)	156	±95% CI ( %)	
**Advance care planning**					
Doing less or a lot less^c^	24 (7.2%)	4.6 to 10.1	4 (2.6%)	0.6 to 5.3	<0.0001
Stayed the same^c^	154 (46.2%)	40.4 to 51.8	49 (31.6%)	24.7 to 38.9	
Doing more or a lot more^c^	155 (46.5%)	40.9 to 52.2	102 (65.8%)	58.1 to 72.8	
*Total*	*333*		*155*		
**Anticipatory prescribing**					
Doing less or a lot less	7 (2.9%)	0.9 to 5.4	3 (1.9%)	0.0 to 4.5	0.82
Stayed the same	98 (41.2%)	34.1 to 48.2	65 (41.9%)	34.3 to 50.0	
Doing more or a lot more	133 (55.9%)	49.1 to 62.8	87 (56.1%)	48.2 to 63.7	
*Total*	*238*		*155*		
**Symptom management**					
Doing less or a lot less	5 (1.4%)	0.3 to 2.7	4 (2.6%)	0.6 to 5.3	<0.0001
Stayed the same^c^	141 (38.3%)	33.8 to 43.5	108 (69.2%)	62.0 to 76.4	
Doing more or a lot more^c^	222 (60.3%)	55.0 to 65.0	44 (28.2%)	21.0 to 35.4	
*Total*	*368*		*156*		
**Support for family members and carers**					
Doing less or a lot less	14 (3.7%)	1.8 to 5.5	8 (5.1%)	1.8 to 8.6	0.01
Stayed the same^c^	116 (30.4%)	25.9 to 35.2	73 (46.8%)	39.5 to 54.5	
Doing more or a lot more^c^	252 (66.0%)	61.2 to 70.5	75 (48.1%)	40.4 to 55.5	
*Total*	*382*		*156*		
**Bereavement support**					
Doing less or a lot less	31 (8.5%)	5.9 to 11.4	15 (9.7%)	5.2 to 14.6	<0.0001
Stayed the same^c^	147 (40.5%)	35.4 to 45.5	96 (61.9%)	54.1 to 69.2	
Doing more or a lot more^c^	185 (51.0%)	45.7 to 56.0	44 (28.4%)	21.8 to 35.9	
*Total*	*363*		*155*		
**Death verification**					
Doing less or a lot less^c^	11 (3.7%)	1.7 to 6.0	59 (38.1%)	30.5 to 46.1	<0.0001
Stayed the same^c^	98 (32.7%)	27.0 to 38.1	69 (44.5%)	36.6 to 52.7	
Doing more or a lot more^c^	191 (63.7%)	58.1 to 69.6	27 (17.4%)	11.7 to 23.5	
*Total*	*300*		*155*		
**Death certification**					
Doing less or a lot less	8 (9.5%)	3.8 to 16.7	15 (9.7%)	5.3 to 14.4	0.52
Stayed the same	54 (64.3%)	52.9 to 74.0	88 (57.1%)	49.4 to 64.9	
Doing more or a lot more	22 (26.2%)	17.4 to 35.8	51 (33.1%)	26.2 to 41.0	
*Total*	*84*		*154*		
**Palliative care for patients who do not have COVID-19**					
Doing less or a lot less	9 (2.4%)	1.0 to 4.1	6 (3.8%)	1.2 to 7.2	<0.0001
Stayed the same^c^	129 (34.3%)	29.8 to 39.1	113 (72.4%)	64.6 to 79.6	
Doing more or a lot more^c^	238 (63.3%)	58.5 to 68.2	37 (23.7%)	17.0 to 30.8	
*Total*	*376*		*156*		
**Collaborative working with specialist palliative care teams**					
Doing less or a lot less	52 (14.1%)	10.5 to 17.7	21 (13.5%)	8.6 to 19.4	<0.0001
Stayed the same^c^	148 (40.1%)	35.3 to 45.3	92 (59.0%)	51.0 to 66.5	
Doing more or a lot more^c^	169 (45.8%)	40.7 to 50.7	43 (27.6%)	20.7 to 34.9	
*Total*	*369*		*156*		

^a^Those who gave the responses, ‘not my role’ and missing responses were excluded from the analysis.^b^χ^2^ test.^c^Pairwise Z-test with Bonferroni correction, significant at <0.05 level.

**Figure 2.**
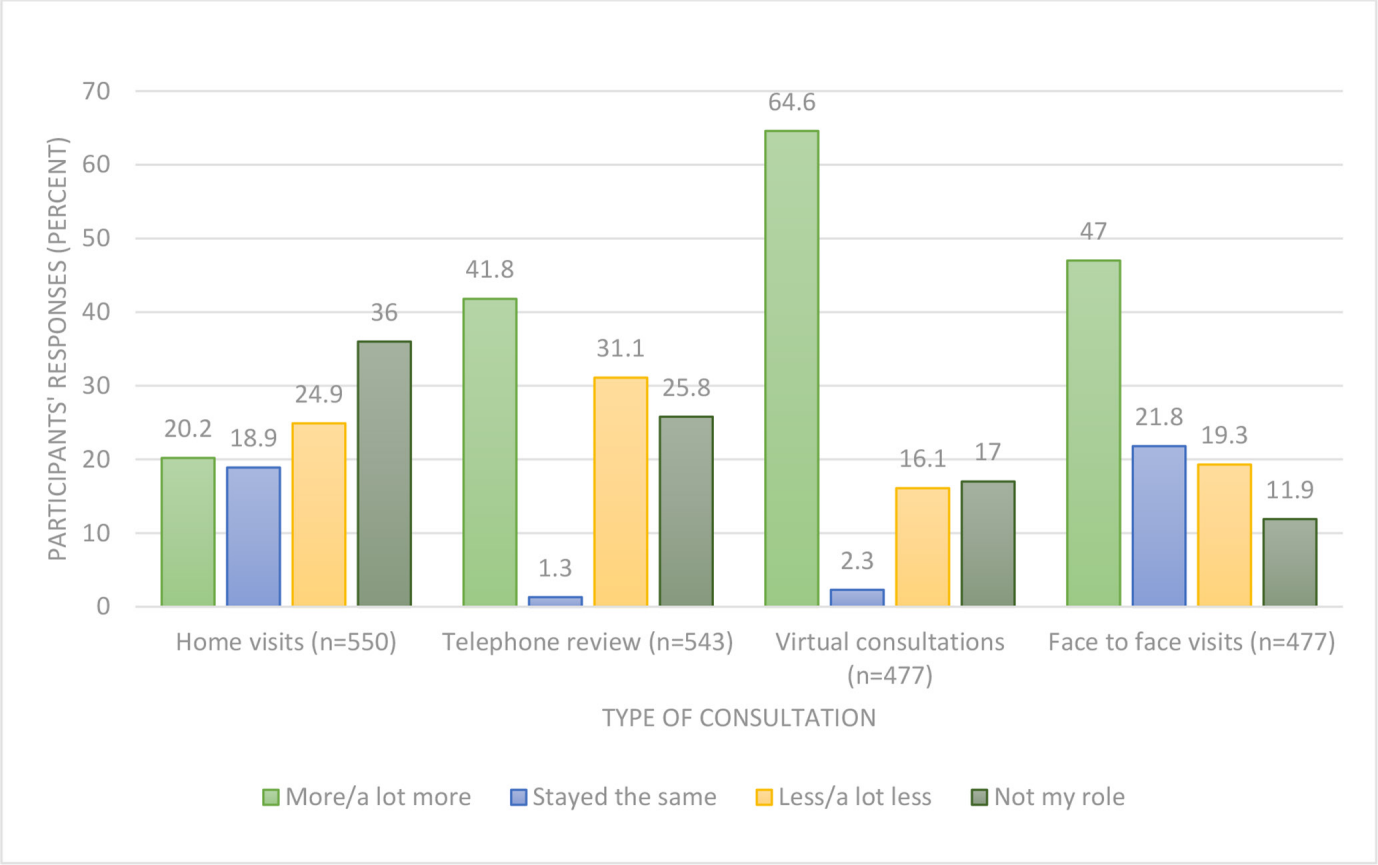
Changes in type of consultation conducted

Both GPs and community nurses reported an increase in consultations (telephone review and virtual consultations, ) related to end-of-life care, but there were changes in the types of consultations undertaken (Figure 2). The greatest increase was in virtual consultations, with 308 (64.6%) undertaking ‘more or a lot more’ of this type of interaction. There was a contrast between the two groups, with over three-quarters (77.0%) of community nurses reporting ‘more or a lot more’ face-to-face visits, whereas almost 40% of GPs reported they were doing ‘less or a lot less’ (*P*<0.0001), as shown in Table 3.

**Table 3.** Comparison between community nurses' and GPs' responses for the type of consultation conducted

	**Please state whether your role in the following areas of palliative care has changed during the COVID-19 pandemic^a^**
Community nurses	±95% CI for (%)	GPs	±95% CI for (%)	*P* value^b^
**Total**					
**Home visits**	387		156		
Doing less or a lot less^c^	97 (44.5%)	38.0 to 50.5	35 (28.9%)	21.0 to 36.9	<0.0001
Stayed the same^c^	36 (16.5%)	11.5 to 21.5	66 (54.5%)	45.1 to 63.2	
Doing more or a lot more^c^	85 (39.0%)	32.6 to 45.9	20 (16.5%)	10.2 to 23.5	
*Total*	*218*		*121*		
**Telephone reviews**					
Doing less or a lot less^c^	93 (37.2%)	31.1 to 42.9	72 (50.7%)	42.1 to 59.0	*P* = 0.08
Stayed the same^c^	7 (2.8%)	0.8 to 5.2	0 (0.0%)	–	
Doing more or a lot more^c^	150 (60.0%)	54.2 to 66.0	70 (49.3%)	41.0 to 57.9	
*Total*	*250*		*142*		
**Virtual consultations**					
Doing less or a lot less^c^	34 (13.0%)	9.0 to 17.6	42 (34.1%)	25.8 to 43.0	<0.0001
Stayed the same	9 (3.4%)	1.5 to 5.6	2 (1.6%)	0.0 to 4.3	
Doing more or a lot more^c^	218 (83.5%)	78.8 to 88.1	79 (64.2%)	55.2 to 72.6	
*Total*	*261*		*123*		
**Face-to-face visits**					
Doing less or a lot less^c^	38 (13.9%)	10.0 to 18.2	53 (39.8%)	31.8 to 48.0	<0.0001
Stayed the same^c^	25 (9.1%)	5.8 to 12.8	79 (59.4%)	50.8 to 67.6	
Doing more or a lot more^c^	211 (77.0%)	71.7 to 81.9	1 (0.8%)	0.0 to 2.4	
*Total*	*274*		*133*		

^a^Those who gave the responses, ‘not my role’ and missing responses were excluded from the analysis.^b^χ^2^ test. ^c^Pairwise Z-test with Bonferroni correction, significant at <0.05 level.

Both GPs and community nurses, described concerns about remote consultations in end-of-life care. There were descriptions of a loss of *'professional intimacy'* (responder 37, GP partner, England)*,* and frustrations among professionals about the limitations of telephone or virtual contacts with patients:

*'I have also found it frustrating to be giving support by telephone when I would usually be visiting a patient to assess their needs. I am quite sure that some patients have deliberately played down their health issues because they felt that the nurses were too busy already and didn't want to be a burden.'* (Responder 78, community staff nurse, Scotland)

It was felt that telephone and virtual consultations were a cause of confusion and distress for patients, and caused *'huge emotional trauma to families'* (participant 441, district nurse, Northern Ireland):

*'The virtual contact was confusing for some patients and almost became something just to tick the box that you had"seen"them in case they passed away.'* (Responder 498, GP partner, England)

A frequent response from community nursing staff was that while their visits to patients had increased, other services, both general practice and specialist palliative care services, had *'backed away '* leaving them feeling abandoned and vulnerable compared to other colleagues:

*'GPs and other community services have backed away leaving us to deal with a lot of difficult situations and questions from families. I feel the end-of-life care given by GPs has been dangerous and neglectful and has not been seamless and high quality.'* (Responder 129, district nurse, England)

GP responders shared this concern. Some GPs reported continuing to provide regular home visits for end-of-life care, but others described feeling under pressure to decrease their home visits owing to infection control procedures, resulting in them not being able to 'g*ive the support* [to patients, families and nursing colleagues] *I would normally wish to'* (responder 475, sessional GP, England). The need for clinical leadership within the team providing end-of-life care in the community, while not unique to GPs, was compromised by service changes:

*'We were encouraged as GPs not to visit care homes if possible, however our clinical leadership role within the care home setting was missed and when we got back in there it helped to get back to our usual multidisciplinary teamworking … The idea that you can replace that sort of team with technology at a time of great stress was proved wrong.'* (Responder 135, GP partner, Scotland)

Views of support from specialist palliative care services were mixed, with some participants describing a positive opportunity to work more closely with these services in the community. Some experienced support through online communities of practice set up by their local hospice. However, others described difficulties communicating and coordinating care with specialist palliative care colleagues, describing them as distant and again reinforcing the reliance on community nursing staff:

*'Districtnurses have borne the bulk during COVID. They are, and continue to be, amazing. The"specialist"palliative care nurses occasionally raise their heads, but are distant and remote from general practice which is a shame.'* (Responder 67, deputy district nurse team leader, England)

### Theme 3: Fears, repeated loss, and undervaluation of contributions threatens resilience and wellbeing of community healthcare teams

Changes in the delivery of care, team dynamics and roles, and increased involvement in end-of-life care had consequences for the emotional wellbeing of responders. Some described teams pulling together *'*and becoming*'* stronger and more bonded Others described their experiences as emotionally and physically draining. Witnessing high volumes of people dying, and managing anxiety around infection control, were significant factors:

*'*[Nursing] *staff felt accused of bringing the virus into the home, there was some public shaming as well as public support, the issue of* [personal protective equipment] *clouded everything (was there enough, was it being used correctly, who was responsible?) … Deaths were more rapid than flu. Many staff got the virus but thankfully no one seriously … Staff have been left broken and there are symptoms of* [post-traumatic stress disorder]*, depression, and anxiety* *.'* (Responder 148, GP partner, Scotland)

Responders described the impact of staff shortages within the team as a result of colleagues becoming unwell with COVID-19, or having to self-isolate. Some had experienced the death of a colleague from COVID-19. Others expressed fears for their own safety, particularly if they had health concerns of their own:

*'I have been frightened coming to work for the last 5 months as I* [have risk factors], *so a prime candidate for COVID-19. But am proud to say I have not shirked my responsibility and have done my duty. It has been very difficult especially when rushing to someone poorly’s house and having to doff and don* [personal protective equipment] *before seeing the patient.'* (Responder 149, district nurse, England)

Support from colleagues, including nursing team leaders, was highly valued, although formal psychological support was lacking. Many responders described feeling that, as community and primary care staff, their contributions to patient care during the first wave of the pandemic did not receive the same attention as hospital care owing to a *'concerning focus on critical care capacity in hospital'* (responder 1, sessional GP, England). This had a negative impact and contributed to a perception that they were undervalued:

*'….the general public seemed to think COVID-19 only existed in hospitals and not in people’s homes. Care homes were forgot about and they seemed to have little or no guidance in relation to infection prevention.'* (Responder 200, district nurse, England)

## Discussion

### Summary

During the first wave of the COVID-19 pandemic, community nurses and GPs experienced a substantial increase in the need for and complexity of palliative and end-of-life care. Specific palliative care activities increased, with community nurses taking greater responsibility in most areas of care, including symptom control and the provision of support to family members. GPs reported an increase in advance care planning. Working hours changed to meet rising demands for care at home through a mainly ad hoc approach.

Changes in the mode of service delivery, including increased virtual consultations, resulted in reports of disconnection within and between teams. Community nursing team members particularly described a sense of abandonment and perceived that other services, including general practice and specialist palliative care, had withdrawn. GPs reported feeling that the utility of virtual consultations was limited in the end-of-life care context. A significant emotional toll was experienced owing to the impact of providing care during the pandemic, with fears relating to uncertainty and loss of the usual mechanisms of interdisciplinary and collegial support.

### Strengths and limitations

This is the first UK-wide survey undertaken to understand the impact of the COVID-19 pandemic on healthcare services involved in the provision of community-based end-of-life care. It provides valuable insights into the role of primary healthcare and the findings are highly relevant to practice, service delivery models, and policymaking as the pandemic progresses.

The minimum number of returns was achieved, but the response rate was low among GPs even though the survey was distributed through well-respected professional organisations. This may be owing to the timing of the survey, when workload related to the pandemic was high and that there were a large number of other surveys taking place concurrently. The findings are likely to reflect the views of primary care professionals with an active role or interest in palliative care, and may not be representative of the wider population of GPs and community nurses.

### Comparison with existing literature

There is a lack of previous research to inform practice, service delivery, or policy,^9,16^ despite the importance of the provision of high-quality end-of-life care in a pandemic.^17,18^ Responders in this study perceived that their role in the pandemic response has received less focus than the response of hospital care.^19,20^ Primary healthcare teams have a pivotal role in end-of-life care, and feeling undervalued may have contributed to the significant emotional distress experienced during the first wave of the COVID-19 pandemic.

The increased need for palliative and end-of-life care in the community is in keeping with research from previous pandemics and is borne out in COVID-19 population data.^1,21^ The increase in deaths in the community has placed extra time and resource pressures on both GPs and community nurses, which were often met through the efforts of highly committed individual healthcare professionals.^19^ Much of the increase in care was for patients dying of conditions other than COVID-19, as a result of patient choice to stay at home, particularly with visiting restrictions in hospitals.^22^ The resulting increase in delivery of end-of-life care and management of complexity in the community contrasts with fears at the start of the pandemic that end-of-life care would be required for a large number of people dying with COVID-19, and admission would have to be avoided owing to such significant pressure on hospital beds, including critical care.

Community nurses have previously described end-of-life care as one of the most rewarding aspects of their job.^23^ This research provides insights into new responsibilities, rapidly assumed by community nurses in almost every aspect of end-of-life care during the pandemic. The context for the delivery of end-of-life care in the community changed drastically with national lockdowns, self-isolation, and shielding resulting in patients at home alone in the community, unable to access their usual social support. Community nurses represent one of the few professional groups with whom people at the end of life had face-to-face contact during the pandemic. The level of skill, compassion, and resilience required to undertake this task must be recognised and valued.^24^

Previous research has exposed tensions in relationships both within primary healthcare teams, and between these teams and specialist palliative care colleagues.^25^ Increased use of virtual consultations by GPs and specialist palliative care teams during the first wave of the pandemic appeared to exacerbate such conflicts, with community nurses describing a feeling of abandonment and isolation. The interpretation of policies recommending that home visits by GPs should be limited as far as possible^26^ may have contributed to this situation. GP responders in this survey and elsewhere have specifically highlighted the importance of face-to-face home visits as an ongoing priority for care of vulnerable patients in the community, including those who are dying.^27,28^ They described a sense of moral distress related to the change in their role associated with increased use of remote consultation. The role of GPs working alongside community nurses in the provision of end-of-life care not only involves specific clinical tasks, but also reviewing, affirming, and supporting care decisions within the multidisciplinary team. This is particularly relevant in the context of COVID-19 as a new condition, where clinicians must take responsibility for complex and nuanced clinical decisionmaking, and collectively manage a great deal of clinical risk and uncertainty.^29^

Providing care for the dying during a pandemic has a profound emotional impact on staff. Access to training, support, and debrief have been identified as important aspects of a pandemic response in previous pandemics,^30^ and remain relevant now.

### Implications for practice and research

The findings of this survey highlight the impact on both services and individuals providing end-of-life care during the first phase of the COVID-19 pandemic. Opportunities and potential unintended consequences in the use of virtual technology for remote consultations with patients at the end of life and their families must be better understood if this practice is to continue. Furthermore, the potential of technology to improve interprofessional communication requires further investigation. Understanding the perspectives of patients and families would be valuable.

Future research should include more in-depth investigation into specific aspects of end-of-life care such as advance care planning and end-of-life care in care homes. This further research is required in the context of a constantly evolving pandemic situation and changing knowledge of the management of COVID-19, which has a direct impact on care decisions including hospital admissions.

There is an immediate need for policymakers and commissioners to recognise the sustained increased need for end-of-life care in the community and the critical role of primary healthcare services in the delivery of this care. The findings of the survey suggest a disconnect between teams involved in end-of-life care in the community and a need to rebuild trusted relationships through truly integrated approaches between GPs, community nurses, and specialist palliative care services. Policy guidance and service models must place focus on and support the multidisciplinary team relationships that are necessary to deliver this care most effectively. Current guidance relating to the roles of specific services has the potential to fragment teams. Ensuring support for individuals involved in the provision of this care, through team relationships, training opportunities, and debrief also requires attention.

This study has identified the contrasting and potentially conflicting roles that emerged between GPs and community nurses in their responses to the increased demand for and complexity of palliative and end-of-life care in the community in the early months of the COVID-19 pandemic. The significant emotional impact, especially for community nurses, needs to be addressed alongside promoting effective, collaborative, and mutually supportive teamworking that can recognise and quickly adapt to changing patient needs.

## References

[1] Office for National Statistics Coronavirus (COVID-19): Latest data and analysis on coronavirus (COVID-19) in the UK and its effect on the economy and society. https://www.ons.gov.uk/peoplepopulationandcommunity/healthandsocialcare/conditionsanddiseases.

[2] Graham NSN, Junghans C, Downes R (2020). SARS-CoV-2 infection, clinical features and outcome of COVID-19 in United Kingdom nursing homes. J Infect.

[3] Raleigh V (2020). Deaths from Covid-19 (coronavirus): how are they counted and what do they show?. https://www.kingsfund.org.uk/publications/deaths-covid-19.

[4] Bone AE, Finucane AM, Leniz J (2020). Changing patterns of mortality during the COVID-19 pandemic: population-based modelling to understand palliative care implications. Palliat Med.

[5] British Medical Association,, Royal College of General Practitioners (2021). COVID-19 workload prioritisation unified guidance. https://www.bma.org.uk/media/3654/bma-rcgp-covid-workload-prioritisation-nov-2020.pdf.

[6] NHS England (2020). COVID-19 prioritisation within community health services.

[7] Ahmedzai SH, Dickman A, Nwosu AC (2020). Letter: rapid response: managing COVID-19 symptoms in the community (including at the end of life): NICE NG163 is a welcome step, but needs review. BMJ.

[8] Patel MS, Phillips CB, Pearce C (2008). General practice and pandemic influenza: a framework for planning and comparison of plans in five countries. PLoS One.

[9] Mitchell S, Maynard V, Lyons V (2020). The role and response of primary healthcare services in the delivery of palliative care in epidemics and pandemics: a rapid review to inform practice and service delivery during the COVID-19 pandemic. Palliat Med.

[10] Higginson I, Murtagh F, Preston N CovPall Study. Improving palliative care for people affected by the COVID-19 pandemic by sharing learning — the national and international response. https://www.kcl.ac.uk/cicelysaunders/research/evaluating/covpall-study/covpall-study.

[11] von Elm E, Altman DG, Egger M (2008). The Strengthening the Reporting of Observational Studies in Epidemiology (STROBE) statement: guidelines for reporting observational studies. J Clin Epidemiol.

[12] Johnson H, Brighton LJ, Clark J (2020). Experiences, concerns, and priorities for palliative care research during the COVID-19 pandemic: a rapid virtual stakeholder consultation with people affected by serious illness in England.

[13] Mitchell S, Loew J, Millington-Sanders C, Dale J (2016). Providing end-of-life care in general practice: findings of a national GP questionnaire survey. Br J Gen Pract.

[14] Braun V, Clarke V (2006). Using thematic analysis in psychology. Qual Res Psychol.

[15] Burnard P, Gill P, Stewart K (2008). Analysing and presenting qualitative data. Br Dent J.

[16] Bowers B, Pollock K, Oldman C, Barclay S (2021). End-of-life care during COVID-19: opportunities and challenges for community nursing. Br J Community Nurs.

[17] Luker KA, Austin L, Caress A, Hallett CE (2000). The importance of 'knowing the patient': community nurses' constructions of quality in providing palliative care. J Adv Nurs.

[18] Ramanayake RPJC, Dilanka GVA, Premasiri LWSS (2016). Palliative care; role of family physicians. J Family Med Prim Care.

[19] Jaakkimainen RL, Bondy SJ, Parkovnick M, Barnsley J (2014). How infectious disease outbreaks affect community-based primary care physicians: comparing the SARS and H1N1 epidemics. Can Fam Physician.

[20] Cinti SK, Wilkerson W, Holmes JG (2008). Pandemic influenza and acute care centers: taking care of sick patients in a nonhospital setting. Biosecur Bioterror.

[21] Fleming DM (1996). The impact of three influenza epidemics on primary care in England and Wales. Pharmacoeconomics.

[22] Office for National Statistics (2021). Deaths registered weekly in England and Wales, provisional: week ending 15 January 2021. https://www.ons.gov.uk/peoplepopulationandcommunity/birthsdeathsandmarriages/deaths/bulletins/deathsregisteredweeklyinenglandandwalesprovisional/weekending15january2021.

[23] Walshe C, Luker KA (2010). District nurses' role in palliative care provision: a realist review. Int J Nurs Stud.

[24] Green J, Doyle C, Hayes S (2020). COVID-19 and district and community nursing. Br J Community Nurs.

[25] Burt J, Shipman C, White P, Addington-Hall J (2006). Roles, service knowledge and priorities in the provision of palliative care: a postal survey of London GPs. Palliat Med.

[26] NHS England (2020). Guidance and standard operating procedures. General practice in the context of coronavirus (COVID-19), Version 2.1. https://web.archive.org/web/20200416164850/https://www.england.nhs.uk/coronavirus/wp-content/uploads/sites/52/2020/03/C0133-COVID-19-Primary-Care-SOP-GP-practice_V2.1_6-April.pdf.

[27] Macdonald G, Vernon G, McNab D, Murdoch JC (2020). Home visits for vulnerable older people: journeys to the 'Far End'. Br J Gen Pract.

[28] Mitchell S, Hillman S, Rapley D (2020). GP home visits: essential patient care or disposable relic?. Br J Gen Pract.

[29] Rutter H, Wolpert M, Greenhalgh T (2020). Managing uncertainty in the covid-19 era. BMJ.

[30] Campbell C, Baernholdt M (2016). Community health workers' palliative care learning needs and training: results from a partnership between a US university and a rural community organization in Mpumalanga Province, South Africa. J Health Care Poor Underserved.

